# Glycocalyx Disintegration Is Associated with Mortality in Chronic Heart Failure

**DOI:** 10.3390/jcm14103571

**Published:** 2025-05-20

**Authors:** Patricia P. Wadowski, Martin Hülsmann, Irene M. Lang, Christian Schörgenhofer, Joseph Pultar, Constantin Weikert, Thomas Gremmel, Sabine Steiner, Renate Koppensteiner, Christoph W. Kopp, Bernd Jilma

**Affiliations:** 1 Department of Internal Medicine II, Division of Angiology, Medical University of Vienna, 1090 Vienna, Austria; joseph.pultar@stpoelten.lknoe.at (J.P.); n01308839@students.meduniwien.ac.at (C.W.); thomas.gremmel@mistelbach.lknoe.at (T.G.); sabine.m.steiner@meduniwien.ac.at (S.S.); renate.koppensteiner@meduniwien.ac.at (R.K.); christoph.kopp@meduniwien.ac.at (C.W.K.); 2 Department of Medicine II, Division of Cardiology, Medical University of Vienna, 1090 Vienna, Austria; martin.huelsmann@meduniwien.ac.at (M.H.); irene.lang@meduniwien.ac.at (I.M.L.); 3 Department of Clinical Pharmacology, Medical University of Vienna, 1090 Vienna, Austria; christian.schoergenhofer@meduniwien.ac.at (C.S.);; 4 Department of Anesthesia and Intensive Care Medicine, Universitätsklinikum St. Pölten, 3100 St. Pölten, Austria; 5 Department of Internal Medicine I, Cardiology and Intensive Care Medicine, Landesklinikum Mistelbach-Gänserndorf, 2130 Mistelbach, Austria; 6 Institute of Cardiovascular Pharmacotherapy and Interventional Cardiology (Karl Landsteiner Society), 3100 St. Pölten, Austria; 7 Karl Landsteiner University of Health Sciences, 3500 Krems an der Donau, Austria

**Keywords:** glycocalyx, microcirculation, capillaries, cardiomyopathy, mortality

## Abstract

**Background:** Glycocalyx disintegration is associated with adverse outcomes in patients with trauma or sepsis. As microvascular dysfunction has an impact on disease progression in chronic heart failure (CHF) patients, we hypothesized that changes in microcirculation might be associated with mortality. **Methods:** Fifty patients with ischemic and non-ischemic cardiomyopathy and conservative treatment with baseline measurements of the sublingual microcirculation (via Sidestream Darkfield videomicroscopy) were followed up for two years. Glycocalyx thickness was assessed indirectly by calculation of the perfused boundary region (PBR). **Results:** Loss of glycocalyx was pronounced in non-survivors after one, n = 10, and two years, n = 16; PBR: 2.05 μm (1.88–2.15 μm) vs. 1.87 μm (1.66–2.03 μm) and 2.04 (1.93–2.11) vs. 1.84 (1.62–1.97); *p* = 0.042 and *p* = 0.003, respectively. Area under the ROC curve for the analysis of the predictive value of PBR on two-year mortality was 0.77 (*p* = 0.003; SE: 0.07, CI (95%): 0.63–0.91). ROC curve analysis determined a PBR of 1.9 μm as the best predictor for two-year mortality (sensitivity: 0.81; specificity: 0.59). Moreover, multivariate regression analysis revealed PBR and functional capillary density as significant predictors of two-year mortality, *p* = 0.036 and *p* = 0.048, respectively. **Conclusions:** Glycocalyx disintegration is related to poor overall survival in CHF patients.

## 1. Introduction

The disturbance of microcirculation promotes the progression of cardiovascular diseases [1,2]. The glycocalyx has a key role in endothelial protection, and its disintegration is often associated with local as well as systemic inflammatory processes resulting in atherosclerosis [3,4]. Glycocalyx impairment facilitates tissue infiltration by monocytes/macrophages, polymorphonuclears, and lymphocytes [5]. Further, glycocalyx disintegration promotes the formation of tissue oedema, including the myocardial tissue [6,7,8,9]. The increased myocardial water content restricts left ventricular contractility, cardiac output, and diastolic cardiac function [7,10,11]. To date, glycocalyx disintegration is regarded more and more as a crucial mechanism in the development and progression of heart failure [12,13].

Negatively charged proteoglycans are the main components of the glycocalyx and consist of a core protein covalently linked to glycosaminoglycans (GAGs) [14]. The latter are increased in the human plasma during conditions of septic shock [15,16], and of those, hyaluronic acid and heparan sulphate are higher in non-survivors [15]. Another component of the glycocalyx, syndecan-1, was measured as a marker for glycocalyx disintegration in patients with acute decompensated heart failure admitted to the hospital and was predictive of the development of acute kidney injury and mortality [17]. Furthermore, in trauma patients, higher levels of circulating syndecan-1 were associated with increased coagulopathy and mortality [18].

Glycocalyx degradation is promoted by different enzymes like matrix metalloproteinases (MMPs), heparinase, hyaluronidase, a disintegrin and metalloproteinase (ADAM), and N-deacetylase-N-sulfotransferase 1 [19]. The glycocalyx is characterized by a negative charge, which regulates endothelial barrier properties by allowing for selective passage of differently charged molecules [9].

Glycocalyx destabilization, conformational changes, and shedding are the lesion-predilecting processes of thromboinflammation, immunothrombosis, and atherosclerosis [20,21,22,23]. Herein, reactive oxygen species (ROS) and immune cells can interact more readily with the endothelium [24,25]. ROS trigger NLRP3 inflammasome activation, which in turn promotes caspase-mediated pyroptosis [26]. Inflammation also triggers platelet activation and platelet–leukocyte interactions, ultimately resulting in ETosis and (micro-) thrombosis [27].

Besides oxidative stress and inflammation, factors contributing to glycocalyx disintegration include high sodium or glucose levels, hypertension, and lipids [19]. Hypercholesterolemia is linked to disturbed glycocalyx, and the latter can be (partially) restored by statin treatment [28]. In addition, higher HDL levels have a protective effect on glycocalyx properties [29].

The importance of an intact endothelial surface layer has become more and more evident in patients with critical illness, where loss of glycocalyx is linked to an adverse patient outcome [30,31].

In heart failure, syndecan-1 levels correlate inversely with left ventricular ejection fraction, signifying the association of glycocalyx degradation with a more advanced disease state [32]. Moreover, the circulating glycocalyx component heparin sulfate was related to a higher all-cause mortality in heart failure patients with reduced ejection fraction [33].

To date, little data exist with in vivo measurements in patients with chronic heart failure. Previously, we described sublingual microvascular rarefaction in patients with chronic heart failure and optimized guideline-directed medical therapy [34]. Indeed, glycocalyx dimensions, as measured via in vivo sublingual capillaroscopy, did not differ between patients and healthy controls [34]. However, given the importance of glycocalyx dimensions on patient outcome in critical illness and infections [35], we hypothesized that the perfused boundary region, as measured in vivo as an indirect marker of glycocalyx dimensions, might be related to patient mortality in a long-term follow up. The aim of this study was to assess patient survival at one and two years of a previously published CHF patient group [34] and compare microcirculatory and laboratory baseline parameters between survivors and non-survivors of this follow up.

## 2. Methods

We performed a follow up of a previously published cross-sectional mono-center clinical trial [34]. This study was performed in accordance with the Declaration of Helsinki, and the protocol was approved by the local Ethics Committee of the Medical University of Vienna (EC-number: 1734/2013; date of first decision: 3 September 2013). All participants signed a written informed consent.

All patients had a history of severe systolic chronic heart failure with reduced ejection fraction (HFrEF), with a left ventricular ejection fraction below 40% and symptoms as well as signs of heart failure, documented in accordance with the European Guidelines for Heart failure [36]. In addition, inclusion criteria comprised NT-proBNP levels above 2000 pg/mL in at least 1 of the preceding clinical visits, irrespective of the current NYHA class of the patient [34].

Exclusion criteria included diseases predisposing to or indicative of capillary changes, such as a (congenital) von Willebrand disease [37], a history of gastric or intestinal surgery, gastrointestinal bleeding, as well as active gastrointestinal diseases that predispose to gastrointestinal bleeding (e.g., gastro-duodenal ulcer, Crohn’s disease, ulcerative colitis, and colonic diverticulosis).

The recruitment period lasted from February to November 2015 with a consecutive one- and two-year follow up time.

Mortality status was assessed by the database of the Vienna General Hospital, which is connected to patient files in hospitals of Vienna as well as by telephone follow up and the Austrian statistic agency (Statistics Austria).

### 2.1. Microscope Imaging

In vivo sublingual assessment of the microvasculature was performed using a sidestream darkfield videomicroscope (CapiScope HVCS Handheld Video Capillary Microscope, KK Technology, Honiton, Devon, UK), as previously published [34,38,39], by one person to avoid inter-observer variability.

The camera is provided with light emitting diodes using a wavelength of 525 nm to detect the hemoglobin of circulating red blood cells. The standard lens of the microscope enables a 0.92 μm/pixel magnification in 752 × 480 pixels (field of view: 692 × 442). The software for acquisition and calculation of the perfused boundary region (PBR) is supplied by GlycoCheck BV (Maastricht, The Netherlands), and the detailed methodology was described previously [39,40]. The camera is placed under the tongue near the frenulum, and the software identifies micro-vessels below 30 μm of thickness due to the contrast of red blood cells (RBCs). RBC column widths are measured in at least 3000 vessel segments. The PBR is the most luminal part of the glycocalyx, which allows for limited penetration of the RBCs [41]. It is located at both sides of the RBC column; to determine its properties, the distance between the median RBC column width (P50) and the outer edge of the RBC-perfused luminal part of the glycocalyx (=perfused diameter) is calculated using the following equation: (perfused diameter-median RBC column width)/2. The increase in PBR reflects glycocalyx destruction [40,41,42]. The average PBR of microvessels between 5 and 25 μm in diameter was used for statistical analyses. The PBR is inversely proportional to the glycocalyx [42]. The measurement and analysis system has been shown to achieve reliable results and to date has been used in different clinical studies [41,42,43,44,45,46].

To assess capillary density, the software recognizes all micro-vessels below 30 μm of thickness by determination of the red blood cells against the background. Vascular segments (line markers) are placed every 10 μm of the vessel length. The recording process continues until a minimum of 3000 vascular segments. After the acquisition, on the first frame of each recording session, a total of 21 line markers are placed every 0.5 μm of the vascular segments. Only those vessels with an appropriate contrast of more than 60% of all 21 line markers are considered as functional (=valid perfused) vessels. All perfused vessels are referred to as total capillary density. The RBC filling percentage is calculated by determining the percentage of vessels with RBCs present during the recording session (corresponding to 40 frames per session) [40]. The RBC filling percentage and perfused capillary density are regarded as estimates of microcirculatory perfusion [34,40].

### 2.2. Statistics

Statistical analysis was performed using the Statistical Package for Social Sciences (IBM Corp. Armonk, NY, USA, Released 2012). The median and interquartile range of continuous variables are shown. Nonparametric testing was chosen to handle outliers and skewed distributions. Categorical variables are given as numbers (%). We performed the non-parametric Mann–Whitney U test to detect differences in continuous variables of the baseline characteristics between survivors and non-survivors of the follow up period.

The chi-square test was used to assess differences in categorical variables of the baseline characteristics with regard to one- and two-year mortality. Spearman rank correlation was used to assess correlations between microvascular and laboratory parameters at baseline.

In addition, receiver operating characteristic (ROC) curve analyses were performed including the standard error (SE) and 95% confidence intervals (CIs) and used to graphically depict the relation between mortality and capillary density as well as for the calculation of predictive thresholds for capillary density with respect to mortality.

A multivariate regression analysis was performed to describe the relationship between PBR, functional or total perfused capillary density, and mortality with regard to possible influencing laboratory values (NT-proBNP, creatinine, C-reactive protein, albumin, and alanine aminotransferase).

## 3. Results

Clinical characteristics of the followed patients at one and two years are given in Table 1.

After one year, 10 patients (20%) died, and after two years, 16 patients (32%) died.

At baseline, the PBR was 1.93 μm (1.70–2.06 μm) in the overall study population [34].

There was a significant inverse correlation of the PBR and RBC filling percentage r = −0.916, *p* < 0.001 [34].

The PBR was significantly higher in patients who did not survive the follow up period: PBR: 2.05 μm (1.88–2.15 μm) vs. 1.87 μm (1.66–2.03 μm), *p* = 0.042, after one year and 2.04 μm (1.93–2.11 μm) vs. 1.84 μm (1.62–1.97 μm), *p* = 0.003, after two years, Table 2.

At the 1-year follow up, there was no difference in RBC filling percentage (71% [70–74%] vs. 74% [71–78%], *p* = 0.087) or the functional (2732 μm/mm^2^ [1820–3141 μm/mm^2^] vs. 2407 μm/mm^2^ [2085–2736 μm/mm^2^], *p* = 0.369) or total perfused capillary density (3525 μm/mm^2^ [2410–6435 μm/mm^2^] vs. 3538 μm/mm^2^ [3043–4497 μm/mm^2^], *p* = 0.971) between survivors and non-survivors, Table 2.

Non-survivors at the 2-year follow up had a significantly lower RBC filing percentage, signifying disturbed microcirculatory perfusion (71% [70–74%] vs. 75% [71–79%], *p* = 0.028). There was no difference in functional (2630 μm/mm^2^ [2028–2974 μm/mm^2^] vs. 2403 μm/mm^2^ [2068–2688 μm/mm^2^], *p* = 0.3) or total perfused capillary density (3568 μm/mm^2^ [2963–5339 μm/mm^2^] vs. 3538 μm/mm^2^ [3021–4397 μm/mm^2^], *p* = 0.75) between non-survivors and survivors, Table 2.

As reported previously, at baseline, PBR correlated with inflammation markers (fibrinogen: r = 0.58, and C-reactive protein: r = 0.42), platelet count (r = 0.36), and measures of renal/liver function such as estimated glomerular filtration rate (r = −0.34), total bilirubin (r = −0.38), and albumin (r = −0.30) in CHF patients, all *p* < 0.05 [34]. In addition, these inflammatory markers correlated with the RBC filling percentage (fibrinogen: r = −0.66, *p* < 0.001; C-reactive protein: r = −0.48, *p* < 0.001; platelets: r = −0.39, *p* = 0.007; albumin: r = 0.26, *p* = 0.085). The leukocyte count did not correlate with the microvascular parameters [34].

Moreover, there was also a positive correlation with the inflammatory marker fibrinogen-to-albumin ratio with PBR at baseline (r = 0.57, *p* < 0.001) and with RBC filling percentage (r = −0.64, *p* < 0.001).

Of these markers, non-survivors of the one-year follow up had lower baseline levels of alanine aminotransferase, *p* = 0.019, Table 1. The other parameters did not differ between survivors and non-survivors at the one-year follow up, Table 1.

In contrast, non-survivors of the two-year follow up had significantly higher baseline NT-proBNP and creatinine levels, with a lower estimated glomerular filtration rate (GFR) as compared to survivors, Table 1. Furthermore, higher baseline C-reactive protein and lower levels of albumin and alanine aminotransferase were observed, Table 1.

In a multivariate regression model comprising PBR, functional and total capillary density, NT-proBNP, creatinine, C-reactive protein, albumin, and alanine aminotransferase, PBR and functional capillary density remained significantly associated with patient survival at two years, Table 3.

**Figure 1:**
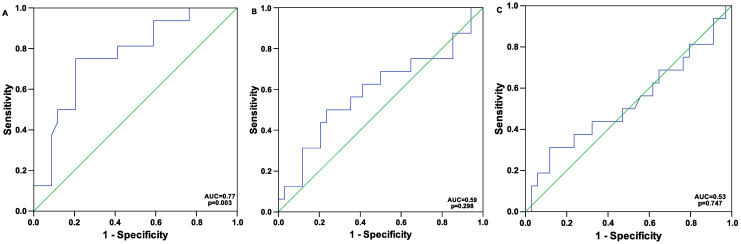
Receiver operating characteristic (ROC) curve for the analysis of the predictive value of (**A**) perfused boundary region (PBR): area under the curve (AUC) = 0.77 ± 0.07 (SE), CI 95%: 0.63–0.91, *p* = 0.003; (**B**) functional capillary density: AUC = 0.59 ± 0.09 (SE), CI 95%: 0.41–0.77, *p* = 0.298; and (**C**) total perfused capillary density: AUC = 0.53 ± 0.09 (SE), CI 95%: 0.34–0.71, *p* = 0.747, depicted as blue line, respectively, for mortality at two years. SE, standard error.

The area under the ROC curve for the analysis of the predictive value of PBR on two-year mortality was 0.77 (*p* = 0.003; SE: 0.07, CI (95%): 0.63–0.91). ROC curve analysis revealed a threshold of 1.9 μm for PBR as the best predictor for two-year mortality (sensitivity: 0.81; specificity: 0.59), Figure 1A.

The area under the ROC curve for the analysis of the predictive value of functional and total capillary density on two-year mortality was 0.59 [*p* = 0.298; SE: 0.09, CI (95%): 0.41–0.77, Figure 1B] and 0.53 [*p* = 0.747; SE: 0.09, CI (95%):0.34–0.71, Figure 1C], respectively.

## 4. Discussion

Glycocalyx disintegration is a central component of endothelial dysfunction driving atherosclerosis and cardiovascular diseases [4]. The latter are associated with altered microvascular perfusion and endothelial barrier properties, often related to disease progression and severity [34,38,39,47,48,49]. Glycocalyx destruction precedes endothelial dysfunction destabilizing vascular homeostasis [9,20]. Herein, all components of the Virchow’s triad including endothelial integrity, vascular perfusion, and coagulation are affected [20]. These processes, though often induced by inflammation, promote a pro-inflammatory and pro-coagulative state eventually leading to tissue oedema, thrombosis, necrosis, and also atherosclerosis [9,50]. Moreover, as endothelial function and vascular integrity is disturbed, glycocalyx degradation promotes the progression of cardiovascular diseases [51].

The presented long-term follow up of our preliminary study examining CHF patients shows the significant association of glycocalyx destruction with mortality, despite guideline-directed OMT. In contrast, we previously could not distinguish an influence of the glycocalyx constitution on mortality in CHF patients with VAD therapy [38]. The observed difference might be due to altered hemodynamics and their possible influence on the glycocalyx in VAD patients or possibly due to benefits/complications associated with the mechanical circulatory support itself [38,52].

In addition to increased glycocalyx disruption, patients who died during the follow up of our study also had higher markers of inflammation. This observation corresponds to the concept of a pro-inflammatory state affecting glycocalyx integrity with an impact on adverse events [4,53]. The latter might occur primarily in the microvasculature, often leading to difficult diagnostic processes, limiting patients’ quality of life, and eventually promoting disease progression. Inflammation is known to directly impact patients’ outcome in CHF [9,54]. This could be shown for C-reactive protein, as higher levels are related to a worse prognosis in patients with acute and chronic heart failure [55,56].

Also, IL-6 was found to be associated with a worse clinical status in CHF and was an independent predictor of mortality in CHF [57]. Other cytokines, such as IL-1ß and IL-18, are associated with inflammation and fibrosis and therefore determine patients’ outcome [58,59,60,61].

Fibrinogen, an acute phase protein [62], has been shown to be independently related to reduced myocardial systolic function [63]. Another parameter, albumin, is documented to impact the incidence and prognosis of heart failure [64,65,66]. CHF patients have lower levels of serum albumin; however, albumin infusions are associated with elevated in-hospital mortality in critically ill patients, which also might be due to disease severity [67].

Recently, the fibrinogen-to-albumin ratio has emerged as a new and easily obtainable marker, which is highly related to survival in CHF [68]. This could be shown in 916 heart failure patients with reduced or preserved ejection fraction, where a higher fibrinogen-to-albumin ratio was independently linked to all-cause mortality, irrespective of the heart failure subtype [68].

Also, during severe acute respiratory syndrome coronavirus 2 (SARS-CoV-2) infection, glycocalyx degradation and the subsequent inflammation and endothelial dysfunction may be regarded as key pathomechanisms accounting for disease progression and complications [20,35,51,69,70,71,72]. In this context, the shedding of glycocalyx components can be regarded as a main factor accelerating viral entry [20,72]. Endothelial dysfunction and endotheliitis evoked by viral invasion drives thromboinflammation affecting the equilibrium of the Virchow’s triad [20]. Together with changes in plasma viscoelastic properties, microclots occur affecting the perfusion of the capillary network [73]. Moreover, sustained changes in glycocalyx composition contributing to inflammatory and pro-coagulative processes are discussed to imply long-lasting sequelae after COVID-19 infection [9,20,74,75].

The results of our long-term follow up are further in-line with previous reports showing an association between syndecan-1, which was measured as a marker of glycocalyx disruption, and 6-month mortality after acute decompensated heart failure [17]. In this study, Neves et al. investigated 201 patients with acute decompensated heart failure admitted to the emergency department [17]. Herein, syndecan-1 levels correlated with hsCRP, and both were independently related to 6-month mortality [17]. Higher plasma levels of syndecan-1 were further associated with the development of acute kidney injury during the hospital stay [17]. As previously reported, we also observed an inverse correlation between PBR and eGFR in our patient group, signifying the occurrence of more pronounced glycocalyx destruction in patients with lower eGFR levels [34]. Furthermore, non-survivors of the 2-year follow up had lower glomerular filtration rates.

Higher plasma levels of syndecan-1 were also associated with higher all-cause mortality and rehospitalization in HF patients with preserved ejection fraction, signifying the association of glycocalyx degradation with adverse patient outcome [76].

The glycocalyx is key in regulating tissue homeostasis, and its intactness is necessary to maintain the filtration barrier and prevent oedema formation [9,77]. Myocardial oedema formation has been described in heart failure and can be attributed to glycocalyx degradation resulting in microvascular barrier dysfunction [78]. The accumulation of water in interstitial and intracellular compartments evokes cardiomyocyte injury, dysfunction, and in consequence cardiac remodeling [9].

Since the glycocalyx represents a fragile structure and preservation of its properties is demanding, therapeutic options remain mainly experimental.

Herein, concepts targeting inflammatory and pro-coagulative pathways are promising to convey glycocalyx protection [9]. Hitherto, medication like sodium–glucose cotransporter 2 (SGLT-2) inhibitors are recommended in heart failure and statins in hyperlipidemia guidelines and are known to exhibit anti-inflammatory properties [79,80]. Moreover, finerenone, a novel non-steroidal mineralocorticoid receptor antagonist, has been shown to convey glycocalyx structure preservation by inhibition of matrix-metaloproteinase 2/9 activity in early diabetic nephropathy in diabetic rats [81]. This could also be the mechanism associated with the protection against COVID-19-associated adverse events in patients with type 2 diabetes and chronic kidney disease [82].

Additionally, experimental approaches covering preconditioning concepts and agents resembling glycocalyx components are under investigation [9].

Moreover, in vivo diagnostic approaches remain challenging. With the use of intravital sublingual capillaroscopy, patients at risk could be identified, which might benefit from further therapy with regard to glycocalyx preservation or restoration. Further studies addressing this question are warranted.

Our main study limitation is the rather small sample size; however, it depicts a heterogenous cohort of patients with CHF.

## 5. Conclusions

In vivo obtained PBR values as indirect measures of the glycocalyx were independently associated with mortality in a long-term follow up of CHF patients. Moreover, this study also highlights the impact of inflammation on glycocalyx dimensions, which contributes to a worse patient outcome. These observations should provide a cornerstone for further research regarding glycocalyx composition and preservation in health and disease, especially during inflammatory conditions.

## Figures and Tables

**Table 1:** Patients’ characteristics.

Follow Up Period of One Year			
	Overall Death n = 10	Overall Survival n = 40	*p*-Value
Age	69 (62–76)	70 (59–77)	0.952
Sex (m/f)	8/2	36/4	0.384
BMI	29 (24–32)	28 (24–32)	0.574
Fibrinogen (g/L)	4.2 (3.6–4.7)	4.0 (3.6–4.6)	0.700
Leukocytes (×10^9^/L)	7.7 (6.5–9.0)	7.6 (6.3–9.1)	0.849
Platelets (×10^9^/L)	215 (162–255)	200 (180–238)	0.926
C-reactive protein (mg/L)	4.2 (3.1–7.5)	5.3 (1.7–8.9)	0.780
Albumin (g/L)	42.6 (37.3–45.3)	43.4 (40.4–45.3)	0.925
Fibrinogen-to-albumin ratio	8.85 (8.33–13.14)	9.52 (7.93–11.48)	0.741
NT-proBNP (pg/mL)	4005 (2826–7937)	2599 (1527–4549)	0.201
Alanine aminotransferase (μmol/s·L)	**0.28 (0.21–0.32)**	**0.37 (0.28–0.48)**	**0.019**
Aspartate aminotransferase (μmol/s·L)	0.35 (0.28–0.45)	0.43 (0.32–0.50)	0.138
Total bilirubin (μmol/L)	9.9 (5.9–16.9)	12.3 (7.2–19.9)	0.586
Serum creatinine (μmol/L)	115.8 (98.6–333.3)	120.7 (95.3–176.8)	0.432
Estimated glomerular filtration rate (ml/min)	50.2 (17.1–62.4)	51.5 (33.1–73.4)	0.343
	**Follow Up Period of Two Years**		
	**Overall Death n = 16**	**Overall Survival n = 34**	***p*-Value**
Age	74 (65–80)	70 (57–75)	0.134
Sex (m/f)	14/2	30/4	0.941
BMI	28.2 (24.6–31.5)	27.9 (24.1–32.3)	0.803
Fibrinogen (g/L)	4.2 (3.7–5.0)	4.0 (3.5–4.5)	0.174
Leukocytes (×10^9^/L)	7.7 (6.4–9.2)	7.6 (6.1–8.8)	0.542
Platelets (×10^9^/L)	215 (176–244)	196 (178–240)	0.706
C-reactive protein (mg/L)	**7.3 (3.9–1.7)**	**3.5 (1.6–7.9)**	**0.026**
Albumin (g/L)	**41.6 (37.4–44.3)**	**44.2 (40.9–45.9)**	**0.037**
Fibrinogen-to-albumin ratio	9.8 (8.34–13.67)	8.85 (7.7–10.89)	0.162
NT-proBNP (pg/mL)	**4693 (3377–11,425)**	**2202 (1483–4243)**	**0.004**
Alanine aminotransferase (μmol/s·L)	**0.28 (0.23–0.35)**	**0.37 (0.28–0.49)**	**0.033**
Aspartate aminotransferase (μmol/s·L)	0.35 (0.28–0.48)	0.43 (0.30–0.50)	0.275
Total bilirubin (μmol/L)	10.6 (7.4–16.9)	12.2 (6.8–20.3)	0.881
Serum creatinine (μmol/L)	165 (108–294)	113 (95–151)	0.066
Estimated glomerular filtration rate (mL/min)	**38.8 (19.5–56.1)**	**56.8 (42.2–75.8)**	**0.045**

Data are presented as median and IQR.

**Table 2:** Microvascular parameters.

	Follow Up Period of One Year		
	Overall Death n = 10	Overall Survival n = 40	*p*-Value
PBR (μm)	2.05 (1.88–2.14)	1.87 (1.66–2.03)	0.042
RBC filling %	71 (70–74)	74 (71–78)	0.087
Functional capillary density (μm/mm^2^)	2732 (1820–3141)	2407 (2085–2736)	0.369
Total capillary density (μm/mm^2^)	3525 (2410–6435)	3538 (3043–4497)	0.971
Ratio (%)	73 (60–85)	71 (57–76)	0.331
	**Follow Up Period of Two Years**		
	**Overall Death n = 16**	**Overall Survival n = 34**	***p*-Value**
PBR (μm)	2.04 (1.93–2.11)	1.84 (1.62–1.97)	0.003
RBC filling %	71 (70–74)	75 (71–79)	0.028
Functional capillary density (μm/mm^2^)	2630 (2028–2974)	2403 (2068–2688)	0.298
Total capillary density (μm/mm^2^)	3568 (2963–5339)	3538 (3021–4397)	0.747
Ratio (%)	73 (57–78)	71 (57–77)	0.771

Data are presented as median and IQR.

**Table 3:** Multivariate regression analyses.

	One Year			Two Years		
	B	CI	P	B	CI	P
PBR	4.8	0.5–27,684	0.087	5.5	1.4–38,820.5	0.036
Functional capillary density	0.03	1.0–1.1	0.083	0.3	1.0–1.1	0.048
Total capillary density	−0.01	0.98–1.0	0.149	−0.01	0.98–1.0	0.064
NT-proBNP	0	1.0–1.0	0.487	0.0	1.0–1.0	0.489
Creatinine	−0.002	0.98–1.0	0.762	−0.01	0.98–1.0	0.224
C-reactive protein	−0.05	0.4–2.6	0.915	0.3	0.8–2.6	0.285
Albumin	0.1	0.8–1.6	0.448	−0.03	0.8–1.2	0.793
Alanine aminotransferase	−8.3	0.0–5.0	0.101	−4.9	0–2.5	0.099

## Data Availability

Raw data generated and/or analyzed during the current study are available from the corresponding author on reasonable request.
